# Usability of a novel Hounsfield units measurement procedure to quantify intercorporal bone graft remodeling in patients after posterior lumbar interbody fusion: a case series

**DOI:** 10.1186/s13256-024-04655-4

**Published:** 2024-07-24

**Authors:** Joey F. H. Reijmer, Job L. C. van Susante, Moyo C. Kruijt, Maarten J. van Gorp, Lex D. de Jong

**Affiliations:** 1 Department of Orthopaedics, Rijnstate Hospital, Wagnerlaan 55, 6815 AD Arnhem, The Netherlands; 2 Department of Orthopaedics, University Medical Center Utrecht, Heidelberglaan 100, 3584 CX Utrecht, The Netherlands; 3 Department of Radiology, Rijnstate Hospital, Wagnerlaan 55, 6815 AD Arnhem, The Netherlands

**Keywords:** Bone remodeling, Case report, Diagnostic techniques and procedures, Spinal fusion

## Abstract

**Background:**

There is a lack of knowledge about the biological process of intercorporal bone graft remodeling after posterior lumbar interbody fusion surgery and whether this process is associated with changes in back pain and intercorporal fusion status. As an alternative to the commonly used but unreliable fusion criteria, Hounsfield units can be used to quantify biological activity and changes in bone mineral content. However, studies assessing Hounsfield units conducted to date do not provide sufficient details about how the bone grafts were segmented to measure the Hounsfield units to allow for replication, and did not assess individual patient trends in graft changes over time. Using the data of nine patients after posterior lumbar interbody fusion, a novel Hounsfield units measurement procedure was developed and used to explore its usability to quantify the bone graft remodeling process.

**Case details:**

We report a case series of nine patients (six male, three female, mean age 64 years, all Caucasian) who all had computed tomography scans performed at 1 and 2 years after posterior lumbar interbody fusion surgery. Overall, seven out of the nine (78%) cases had a 3–41% increase in their bone grafts’ Hounsfield units between 1 and 2 years after surgery. The cases showed large interindividual variability in their Hounsfield units values over time, which coincided with varying levels of back pain and intercorporal fusion status.

**Conclusion:**

The Hounsfield units measurement procedure used for this case series may be useful to quantify intercorporal bone graft remodeling in patients after posterior lumbar interbody fusion, and may be used as an adjunct diagnostic measure to monitor bone graft remodeling over time. Future research is warranted to explore how to interpret bone graft Hounsfield units-values and Hounsfield units trajectories in light of clinical variables or intercorporal fusion status.

## Background

Lumbar spinal fusion is a surgical procedure that is widely being used for the treatment of a variety of spinal disorders and its incidence is rising [1, 2]. Surgical techniques to achieve fusion vary and consensus regarding a superior technique is lacking [3]. To promote interbody fusion the intervertebral disc is removed and replaced with an autologous or allogenic bone graft [4]. This nonvital bone graft has the ability to remodel into vital bone by going through different phases of bone regeneration [5]. This process is characterized by an increase in bone volume and bone quality and should ultimately lead to bony fusion of the two adjacent vertebrae. When this process fails, a nonunion or pseudarthrosis will occur. This important and well-known complication after spinal fusion affects 5–35% of patients and may lead to complaints of pain and a decrease in functional status [6]. Subsequent revision surgery for symptomatic pseudarthrosis is performed in up to 24% of patients [7].

In daily clinical practice, many surgeons and radiologists use plain radiography or computed tomography (CT) images to assess whether their patients’ intercorporal bone grafts are showing signs of increasing bone volume and quality and if the bone grafts are remodeling toward bony fusion or not. However, interpreting these images remains a serious challenge both in terms of how the bone graft is developing as well as ultimately judging whether vertebrae have fused or not [8–10]. A recent systematic review [11] has highlighted that assessment of intercorporal fusion is challenging, especially since there is no widely accepted definition of interbody fusion. This hinders confirmation of the real presence of solid bony fusion. Until a valid and practical method of assessing interbody fusion is available, quantifying the process of intercorporal bone graft remodeling toward fusion may be the most objective alternative for now.

Bone quality or bone mineral density (BMD) is traditionally measured using dual energy X-ray absorptiometry (DEXA) scans. However, this method is not appropriate for assessing patients’ bone graft BMD after spinal fusion surgery because the implanted instrumentation causes visual artefacts, which may affect BMD values [12]. Hounsfield units (HU), on the other hand, highly correlate with BMD [13] and can be measured from CT-images without interference from these artefacts. The use of HU as a means to assess biological activity and increased bone mineral content after lumbar fusion surgery was first described in the early 2000’s [14, 15]. More recent studies showed that HU can also be used to determine bone quality of vertebral bodies and around the bone fusion site [16, 17], further confirming the clinical utility of assessing bone quality using CT-attenuation in HU [18]. HU may also offer a promising method to objectively quantify intercorporal bone graft expansion or resorption over time, and ultimately assist in determining fusion status more objectively [19]. Unfortunately, the studies conducted to date did not provide sufficient details about how the bone grafts were segmented to measure the HU to allow for replication, and did not assess individual patient trends in bone graft changes over time. Therefore, we set out to develop a detailed and reproducible HU measurement procedure and to explore its usability to quantify the bone graft remodeling process in nine patients after posterior lumbar interbody fusion (PLIF).

## Case details

Available data from nine Caucasian patients that consented to participate in a previous trial [20] was collected for this case series. The characteristics of these cases are summarized in Table 1, cases were treated with single-level PLIF surgery using autologous bone graft that was impacted behind a polyetheretherketone (PEEK) cage to promote interbody fusion. No intra- or postoperative complications nor other adverse events were reported for any of the cases.

**Table 1:** Case characteristics

Characteristic	Case 1	Case 2	Case 3	Case 4	Case 5	Case 6	Case 7	Case 8	Case 9
Age, years	60	55	63	69	54	77	60	72	62
Sex	Male	Female	Female	Female	Male	Male	Male	Male	Male
BMI, kg/m^2^	26.4	25.4	33.6	23.9	27.2	32.4	27.1	30	26.6
Spinal level treated	L5–S1	L5–S1	L4–L5	L5–S1	L5–S1	L4–L5	L4–L5	L5–S1	L5–S1
Spondylolisthesis grade^a^	1	2	1	2	2	1	1	2	1
ASA classification^b^	2	2	2	2	1	2	2	2	1
Duration of surgery, minutes	129	101	203	103	105	203	109	119	145
Length of hospital stay, days	2	4	6	5	6	8	5	4	2
Bone graft HU^c^									
At 1 year after surgery	559	229	267	658	460	346	426	545	546
At 2 years after surgery	383	323	297	869	561	296	485	623	561
Back pain VAS score									
Before surgery	66	87	66	73	38	73	65	67	64
At 1 year after surgery	3	7	19	33	4	53	8	19	57
At 2 years after surgery	3	8	69	65	4	54	10	11	64
ODI score^d^									
Before surgery	38	42	54	62	20	48	26	30	32
At 1 year after surgery	2	4	22	34	14	40	0	12	40
At 2 years after surgery	2	6	28	40	8	48	0	12	44
EQ-5D-5L index^e^									
Before surgery	0.58	0.18	0.35	0.58	0.79	Missing	0.57	Missing	0.36
At 1 year after surgery	0.83	0.87	0.83	0.81	0.76	Missing	Missing	0.72	0.57
At 2 years after surgery	1	0.87	0.68	0.62	1	0.78	0.83	0.74	0.62
Fusion judgement^f^									
At 1 year after surgery	Doubtful fusion	No fusion	Doubtful fusion	Doubtful fusion	Fusion	No fusion	Fusion	Fusion	Fusion
At 2 years after surgery	Fusion	No fusion	Fusion	No fusion	Doubtful fusion	Fusion	Fusion	Fusion	Doubtful fusion

*ASA* American Society of Anesthesiologists physical status classification system, *BMI* body mass index, *EQ-5D-5L* European Quality of Life 5 Dimensions Health Questionnaire, *HU* Hounsfield units, *ODI* Oswestry disability index, *VAS* visual analogue scale

^a^The Meyerding classification classifies translation or slip of one vertebral body on another into five grades: 0–25% is grade 1, 25–50% is grade 2, 50–75% is grade 3, 75–100% is grade 4, and greater than 100% is grade 5

^b^ASA scores range from 1 to 6, with a higher score indicating poorer physical status prior to surgery

^c^HU values reported in this table were calculated using the calibration correction factor

^d^ODI scores range from 0 (no disability) to 100 (most severe disability)

^e^EQ-5D-5L values are anchored at 1 (full health) and 0 (a state as bad as being dead)

^f^The process of intercorporal bone remodeling was judged either as having resulted in fusion (“continuous bony bridge from one vertebra to the other, in the absence of any secondary signs of nonunion, such as fracture or loosening of the screws or rods”), doubtful fusion (“doubt about continuity or quality of the bony bridge”), or nonunion (“definite discontinuity or lack of a fusion mass, as well as obvious indicators of mobility like material failure or apparent pseudarthrosis”)

All cases had a 1-year and 2-year follow-up CT-scan using one of three different scanners (Philips iCT 256, Philips Brilliance 40, Siemens Emotion 16) using different kernels (HNP/Myelo and Trauma protocols), kilovoltage peaks (ranging between 100 and 140) and filter types (A or D), which is known to influence HU outcomes [21]. Because no phantom model was used during the original trial we deemed it necessary to calculate a correction factor on the basis of the accessible anatomical structures also imaged on the cases’ CT scans. Details about this procedure and all the cases’ raw data can be found here: https://data.mendeley.com/datasets/632dx4vb96/5.

### Hounsfield unit measurement procedure

**Fig. 1:**
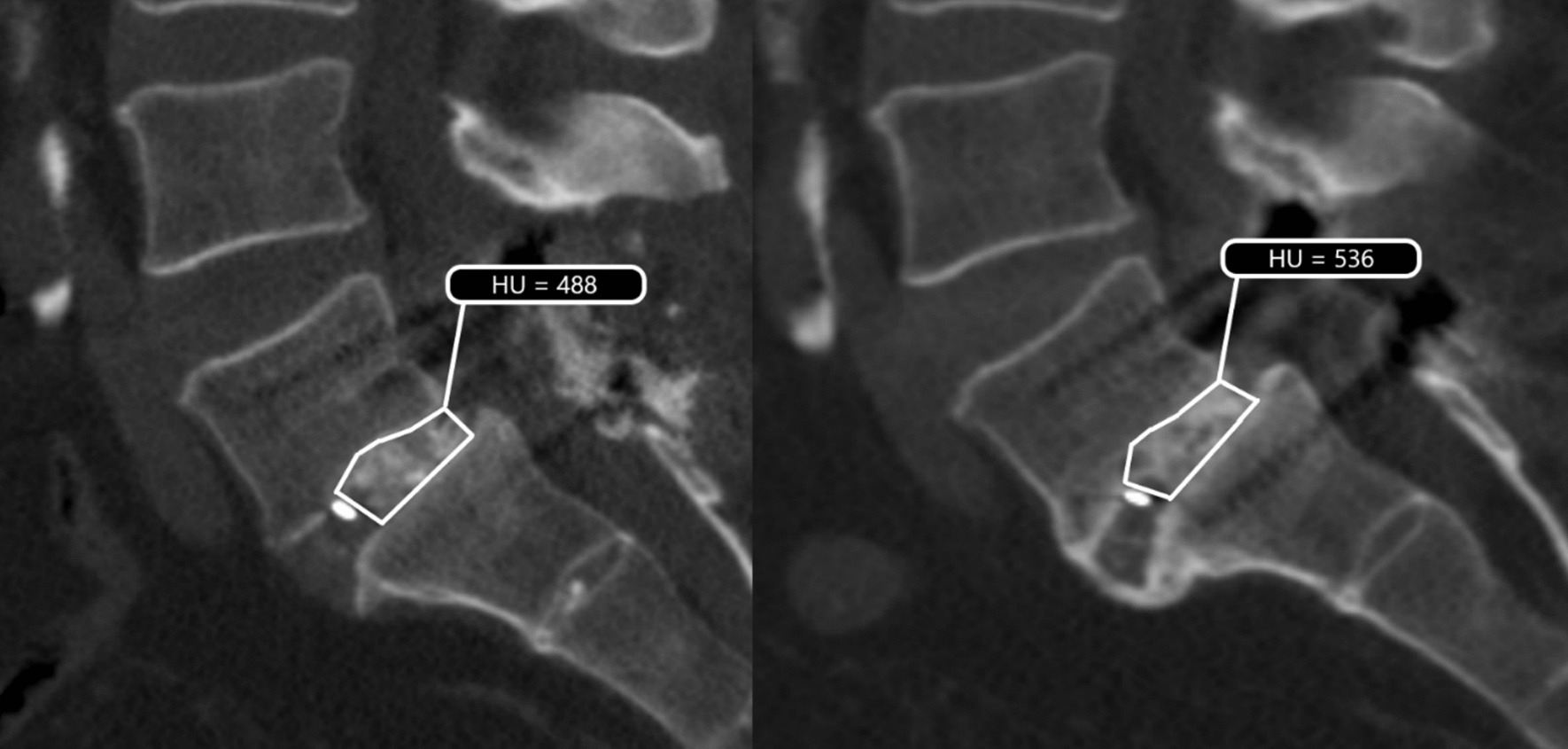
Measuring the intercorporal bone graft’s Hounsfield units of case 8. Freehand drawn regions of interest including the intercorporal bone graft at 1 (left scan) and 2 (right scan) years after surgery. The (0.5 mm) cage markers, which acted as reference points beyond which the regions of interest was demarcated (posteriorly), are clearly visible on both scans. In this case the (raw, uncorrected) HU increased from 488 HU to 536 HU over time

For the HU measurement procedure we used the dynamic oblique multiplanar reformatting option of the Sectra IDS7 software program to position each cases’ CT-scan with the lower endplate of the vertebrae above, and the upper endplate below the grafted area aligned as horizontally as possible. Each image was saved in a 1 mm slice thickness setting and then viewed using the Sectra UniView medical image viewer with the dorsal structures displayed on the right. Subsequently, the observer scrolled through the different sagittal CT-slices to judge in which of these the largest and most hyperintense region of bone graft was visible. This slice was chosen as the primary slice of interest (pSOI) and used as the starting point for all subsequent HU measurements. To ensure that the exact same pSOI could be localized for the intraobserver reliability measurements, as well as for the two-year follow-up CT-scans, the observer scrolled through the sagittal CT-slices from the pSOI until the first available cage marker was visible. This cage marker was used as the reference slice to re-identify the pSOI. Second, using the software program’s freehand drawing option, in the pSOI a region of interest (ROI) was demarcated around the visible area of the cases’ bone graft, resulting in an automatically calculated HU value (see Fig. 1).

Care was taken that the ROI did not include the adjacent lumbar body endplates nor the cage. The latter was ensured by consistently demarcating the ROI posterior to the reference cage marker in all CT-slices used. This exact same procedure was repeated using five adjacent CT-slices to the cases’ right and four to the cases’ left of the pSOI, respectively. Subsequently, the resulting HU values from all CT-slices were summed and divided by the number of each cases’ available CT-slices (typically ten) to obtain a standardized estimate (mean) of the bone graft’s HU both for the 1 year and 2 year CT-scan slices. The 2 year HU outcomes were multiplied with the calculated correction factor. The intraobserver reliability of the measurement procedure was additionally established using 174 CT-slices of the nine cases using their 1-year, and another 174 CT-slices using their 2-year CT scans both measured with a 2 week interval. The resulting intraclass coefficient (ICC) was 0.93 [95% confidence interval (CI) 0.91–0.95, single-rater, consistency, two-way mixed-effects model].

**Fig. 2:**
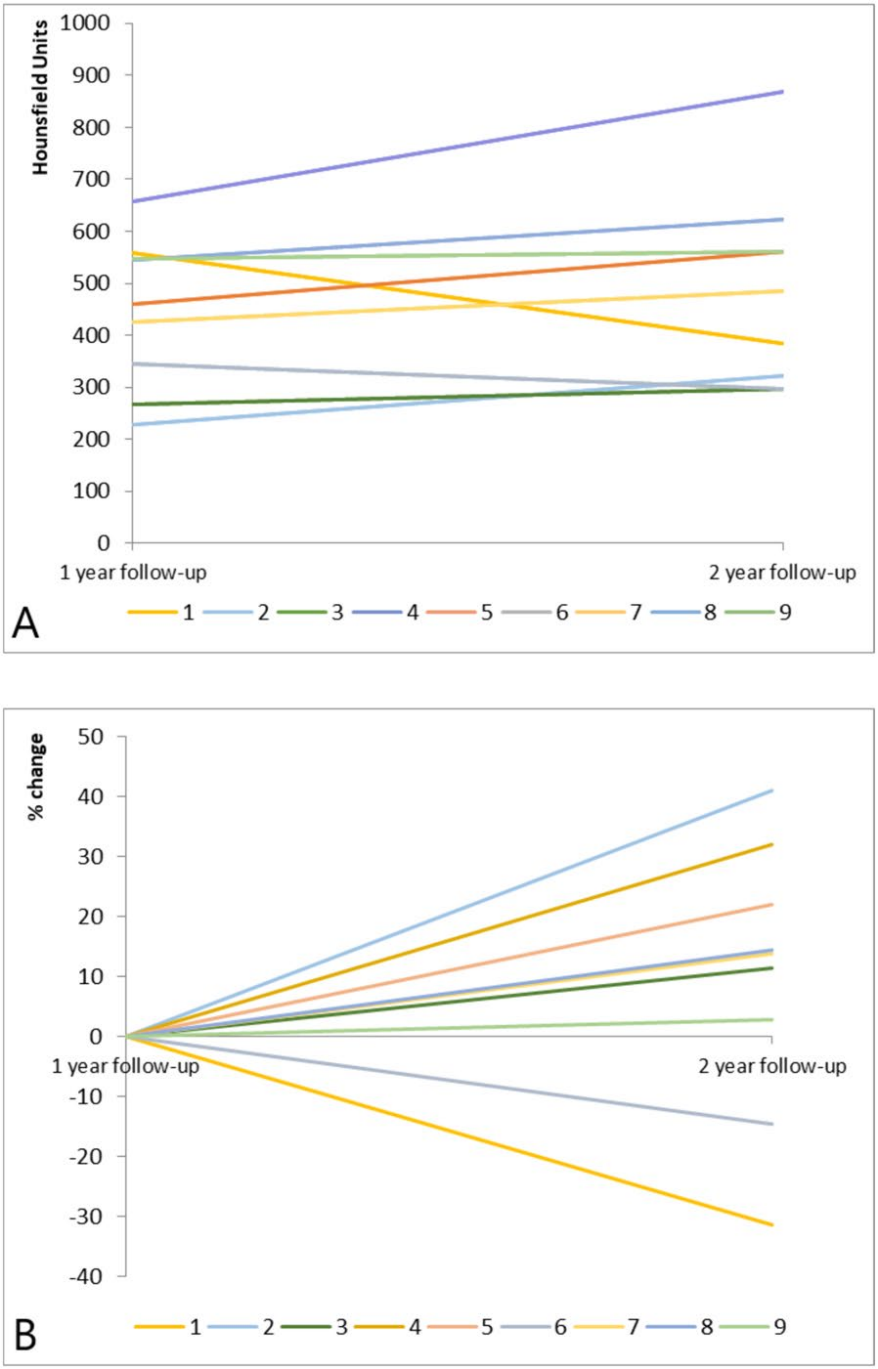
Change in the cases’ bone grafts Hounsfield units over time using internal phantomless calibration. The change in Hounsfield units from 1- to 2-years postoperatively (**A**) and the percentage change, with the first year being the starting point (**B**). Each line represents one individual case

The cases’ changes in bone graft HU values from 1-year to 2-years after surgery are presented in Fig. 2A and their percentage change over time are visualized in Fig. 2B. The correction factors of the cases ranged between 0.66 and 1.49. Overall, there was a wide variety in the cases’ individual postsurgical characteristics, which did not always correspond with each other. For example, one male patient’s (case 8) intercorporal bone graft showed an increase from 545 HU to 623 HU over time (Fig. 1). This patient’s process of bone remodeling coincided with a clear decrease in back pain levels as assessed using a visual analogue scale (VAS) and a decrease in perceived disability related to back pain as assessed using the Oswestry disability index (ODI). Both at 1 and 2 years after surgery, the two vertebrae of this case were subjectively judged as being fused. One female patient (case 2) also showed an increase in HU, with decreases in VAS back pain scores (from 87 to 8) and ODI scores (from 42 to 6) over time, but both at 1 and 2 years after surgery the two vertebrae of this case were judged as not being fused. Overall, seven cases showed an increase in their HU over time, representing a change between 3% and 41%. Two cases showed a negative change of −14% and −31% in their overall HU. One of these was a male patient (case 6) who kept reporting back pain and who showed unchanged ODI scores over time despite the orthopedic surgeon judging his two vertebrae as being fused at 2 years after surgery. The development of the HU of the other male patient (case 1) also suggested that the bone quality was decreasing over time. Nevertheless, this cases’ back pain decreased, his health state as assessed using the European Quality of Life 5 Dimensions Health Questionnaire (EQ-5D-5L) increased notably and his vertebrae were judged as being fused.

## Discussion

Open surgical exploration is the gold standard procedure to confirm lumbar fusion status. However, this procedure is too impractical and considered unethical [11]. As such, quantifying the bone graft remodeling process itself may currently be the only appropriate and reliable method that can inform the orthopedic surgeon about whether their patients’ intercorporal bone grafts are remodeling toward fusion.

Using the data of nine patients after PLIF allowed us to explore the usability of a novel Hounsfield units measurement procedure to quantify intercorporal bone graft remodeling. The excellent intraobserver reliability of the measurement procedure (ICC 0.93) suggested that the measurement procedure was highly reproducible on an individual observer’s basis, and this gave us the confidence to subsequently explore the cases’ bone grafts HU over time. Our cases showed expected large interindividual variability in for example, their HU values, back pain levels, and health status. Overall seven out of the nine (78%) cases had a 3–41% increase in their bone grafts’ HU between 1 and 2 years after surgery.

To our knowledge, only two previous studies have investigated changes in HU over time as a proxy measure of the bone graft remodeling process after spondylodesis. One of these studies [14], published almost 20 years ago, revealed an increase of 23% in HU of the autografts inside fusion cages at 1 year after surgery. Subsequently, a 6% decrease in HU was detected at 2 years. In that study the HU values from all patients were pooled without reporting on baseline differences that affect bone quality, which makes it susceptible to confounding as this could influence the bone grafts’ HU values. In addition, the use of early generation CT scanners may also have influenced the HU values in terms of artefact influence and bias from limitations in scanner calibration. A second study also reported increasing HU-values in individual patients’ intercorporal bone grafts after spondylodesis up to 2 years after spinal fusion surgery [15]. However, in this study single measurements of the participants’ bone graft HU-values were used for pooled trend analysis and details about the scanning procedures were lacking. Therefore, our protocol included a calibration correction factor when different types of CT-scanners were used.

Given the positive association between HU and BMD [13], the increases in HU values of seven of the cases suggest that their bone was still remodeling up to 2 years after surgery, and their increasing bone density may have been a sign of bone graft proliferation toward bony fusion. Conversely, in the two cases (22%) that showed a decrease in their HU, this may have been a sign of bone resorption, which could ultimately lead to fusion failure and pseudoarthrosis. This latter finding is in line with the results of research showing that 5–35% of patients develop pseudoarthrosis after spine fusion surgery [22]. However, for now associations between the downward, upward, or stable trajectories in the bone grafts’ HU and other clinical characteristics, such as back pain, health status and the surgeon’s judgements of fusion status remain elusive. This is illustrated by different cases showing either decreased (for example, cases 2 and 8) or unchanged (for example, cases 3 and 9) back pain levels together with increases in their bone grafts’ HU and health status. Similarly, cases showing clear increases in their bone grafts’ HU were judged as fused (for example case 8) or not fused (for example, case 4), while the two cases (1 and 6) who showed clear decreases in their bone grafts’ HU were both judged as being fused.

The patients’ data presented in this case series have shown that it is currently still unclear how to interpret bone graft HU-values and HU trajectories in light of clinical variables, such as the patients’ level of back pain, perceived disability related to back pain and health status.. Interpretation is also seriously hindered by the unreliability of current fusion judgement criteria [11]. Future research, using much larger patient samples and assessing repeated CT-scans in the first year after PLIF surgery, is needed to gain better insight into the development of HU values over time and thus the process of bone graft remodeling. This will also assist in obtaining more precise and reliable estimates of associations between the different clinical outcomes. Despite this limitation, monitoring the patients’ HU-values over time obtained using our measurement procedure may be a valuable adjunct clinical outcome alongside the unreliable criteria currently used to judge whether this has ultimately led to intercorporal fusion. For example, imagine a patient who is reporting persistent back pain after PLIF surgery and about whom the orthopedic surgeon is unsure about the need for revision surgery given that the patient’s vertebrae are (subjectively) judged as being fused (for example, our cases 3 and 9). Revisiting these patient’s available old and new CT-scans and using these to establish the bone grafts’ HU over time could assist in gauging whether intercorporal bone was indeed expanding, not changing or resorbing over time. In case of clear increases in the patients’ bone graft’s HU, bone remodeling toward fusion would more likely compared with when HU were not changing or even decreasing. In case of the latter situations, the results of additional diagnostics, such as flexion–extension radiographs [23] or the facet fluid sign [24], could be used to further assist in clinical decision-making.

## Conclusion

The Hounsfield units measurement procedure used for this case series may be useful to quantify intercorporal bone graft remodeling in patients after PLIF, and be used as an adjunct diagnostic measure to monitor bone graft remodeling over time. Future research is warranted to explore HU trajectories in light of clinical variables and intercorporal fusion status.

## Data Availability

The datasets used and analyzed during the current study are available and freely accessible at https://data.mendeley.com/datasets/632dx4vb96/5.

## Abbreviations

BMD: Bone mineral density
CT: Computed tomography
HU: Hounsfield units
ICC: Intraclass coefficient
PLIF: Posterior lumbar interbody fusion
